# Antimicrobial Activity of Compounds Isolated from the Nest Material of *Crematogaster rogenhoferi* (Mayr) (Hymenoptera: Formicidae)

**DOI:** 10.3390/insects15121019

**Published:** 2024-12-23

**Authors:** Weihui Bai, Baihe Chen, Huimei Chen, Lei Nie, Mingrong Liang, Yijuan Xu, Yongyue Lu, Lei Wang

**Affiliations:** 1 College of Plant Protection, South China Agricultural University, Guangzhou 510642, China; b2451402768@163.com (W.B.); chenbaiheee@163.com (B.C.); 20222022001@stu.scau.edu.cn (H.C.); nielei98@126.com (L.N.); xuyijuan@scau.edu.cn (Y.X.); luyongyue@scau.edu.cn (Y.L.); 2 School of Biological Sciences, The University of Hong Kong, Hong Kong SAR, China; liangmr0321@connect.hku.hk

**Keywords:** *Crematogaster rogenhoferi*, ant nest extract, antibiosis, GC-MS, immune defense

## Abstract

Ants as social insects live in groups, which increases the risk of contagious diseases. In response to the threat of pathogens, ants have evolved a variety of defense mechanisms, including incorporating antimicrobial chemicals into nest material for nest hygiene. *Crematogaster rogenhoferi* is an arboreal ant, building its nest using plant tissues. It is still unclear how *C. rogenhoferi* is protected against pathogens in its nest. Two main chemicals, 2,2′-methylenebis[6-(1,1-dimethylethyl)-4-methyl-phenol] (MP) and lup-20(29)-en-3-one (LP), isolated from nest materials of *C. rogenhoferi* were used to investigate ants’ anti-pathogenic activity against the entomopathogenic fungus *Beauveria bassiana* and the entomopathogenic bacteria *Serratia marcescens*. The results showed that MP and LP can inhibit the growth of *B. bassiana* through direct contact and fumigation. However, neither MP nor LP had any negative effect on *S. marcescens* growth. Subsequent analysis showed that MP was found in both the abdomen part and the head part of *C. rogenhoferi* workers, and LP was not detected in *C. rogenhoferi* workers. Since LP is a common plant secondary metabolite, it is implied that LP may originate from the plant tissue of *C. rogenhoferi* nest materials. Our results showed that *C. rogenhoferi* capitalizes on its own antimicrobial chemicals and probably the chemical defenses which have evolved in plants to protect itself against pathogens.

## Simple Summary

Ants have evolved several defense mechanisms in response to the threat of pathogenic microorganisms. In this study, we detected two chemicals, 2,2′-methylenebis [6-(1,1-dimethylethyl)-4-methyl-phenol] (MP) and lup-20(29)-en-3-one (LP), from nest materials and evaluated their antimicrobial effects. The results showed that MP and LP significantly inhibited the growth of *Beauveria bassiana* through direct contact and fumigation but did not have any negative effect on *Serratia marcescens* growth. Subsequent analysis showed that MP was found in both the abdomen and the head of *C. rogenhoferi* workers and that LP is likely derived from the plant tissue of *C. rogenhoferi* nest materials. Our result showed that *C. rogenhoferi* capitalize on its own antimicrobial chemicals and probably the chemical defenses which have evolved in plants to protect itself against pathogens.

## 1. Introduction

Ants live in highly connected and densely populated colonies [1], and the tightly socialized colony structure is suitable for the transmission of infectious pathogens [2]. Therefore, ants have evolved a range of defense strategies to counteract infectious diseases, including “social immunity”, e.g., avoidance of pathogenic microbes through mutual grooming behavior [3,4]. The application of antibiotic secretions from glands is very common in ants [5,6]. For instance, the salivary glands of ants secrete antibiotics, antimicrobial peptides, and related digestive enzymes to inhibit the spread of bacteria [7,8]. When individuals are at risk of contagious diseases, ants treat injured nestmates with antimicrobial compounds from metapleural glands to disinfect their wounds [9], as well as injecting infected nymphs with antimicrobial toxins to kill nymphs and pathogenic bacteria in the cocoon before the pathogen spreads [10] or clearing infested ant carcasses and detritus out of the nest [11], which can effectively prevent the further spread and propagation of pathogenic bacteria in the colony.

Many ants build nests. The nest functions as a physical barrier to the intruders and provides a relatively stable condition for the survival and development of the colony. Meanwhile, an ant nest is also an ideal site for microorganism growth because of its suitable and regulated moisture and temperature conditions. To suppress the growth of pathogenic microorganisms inside the nest, fire ants *Solenopsis invicta* Buren (Hymenoptera: Formicidae) use their own volatile substances to fumigate the entire nest to serve this purpose [12]. Ants also secrete antimicrobial compounds and add them to their nests [13,14,15]. Many plants produce antimicrobial substances in their roots, stems, leaves, flowers, fruits, and seeds, exhibiting excellent inhibitory effects on bacteria and fungi [16]. Research has shown that insects employ plants or their secondary metabolite against microorganisms. For example, wood ants *Formica paralugubris* Eichwald (Hymenoptera: Formicidae) incorporate tree resin with antimicrobial properties into their nests to fight pathogens [17,18]. This behavior is also found in honeybees [19,20,21]. Moreover, arboreal ants build hive-like nests in *Myrmecodia* spp. (Gentianales: Rubiaceae) trees, which are rich in antimicrobial substances (e.g., polyphenols and tannins) [22,23].

*Crematogaster rogenhoferi* (Mayr) (Hymenoptera: Formicidae), a common arboreal ant in South China, builds hive-like nests in trees. The nests of *C. rogenhoferi* are mainly composed of plant debris or leaf litter, and the average temperature in nests is 22.35 °C [24]. The nest materials of *C. rogenhoferi* and regulated temperature conditions make *C. rogenhoferi* nests an ideal environment for microorganisms’ growth. To keep nest hygiene, Langshiang and Hajong (2018) found that *C. rogenhoferi* uses actinomycetes against pathogenic fungi growth; seven actinomycetes were isolated from nest materials and show antifungal property against the growth of *Metarhizium anisopliae* (Voronin) (Hypocreales: Clavicipitaceae), *Beauveria bassiana* (Bassi) (Hypocreales: Clavicipitaceae) and *Lecanicillium lecanii* (Zimmermann). (Hypocreales: Cordycipitaceae) [25]. However, rare studies have reported how *C. rogenhoferi* applies chemicals against pathogens. In this study, we analyzed the chemical components of the nest materials and evaluated the effectiveness of the main components on pathogenic fungi and bacteria.

## 2. Materials and Methods

### 2.1. Samples and Chemicals

The *C. rogenhoferi* with nest were collected from the *Acacia auriculiformis* (Cunn) (Fabales: Fabaceae) tree in the woodland of Liantang Reservoir in Shenzhen, China (22°48′38.5″ N 113°55′2.0″ E) on 7 August 2024. A *C. rogenhoferi* colony with its nest were reared in a plastic box (38 cm × 26 cm × 21 cm, volume 15 L) which was coated with a mixture of ethanol and talc powder in its inner wall to prevent workers from escaping [26]. Two colonies were collected for this experiment. The collected colonies were fed with distilled water, 10% *w*/*w* sugar water, and larvae of yellow mealworm *Tenebrio molitor* L. (Coleoptera: Tenebrionidae) under laboratory conditions at 26 ± 2 °C and 60 ± 2% RH, with a 16:8 light to dark cycle maintained.

*Serratia marcescens* B. (Enterobacterales: Enterobacteriaceae) LWCC1044 was selected as the test pathogenic bacterium and purchased from Guangdong Provincial Microbial Strain Preservation Center. Using the dilution spreading method, Luria–Bertani (LB) liquid medium with a concentration of 10^8^ CFU (Colony Forming Unit) of *S. marcescens* was obtained and spread evenly on LB solid medium, and *S. marcescens* used for bioassays were harvested from LB solid medium.

*Beauveria bassiana* GDMCC 3.428 strain were selected as the test pathogenic fungi purchased from Guangdong Provincial Microbial Strain Preservation Center. Conidia for bioassays were obtained from 10-day-old cultures in potato dextrose agar (PDA) plates. Only spores with a germination rate of at least 90% on PDA were used for the bioassays. *B. bassiana* was diluted with 0.5% Tween sterile water to 1 × 10^8^ spores/mL, evenly spread and cultured on PDA solid medium, and *B. bassiana* spores for bioassay were obtained from PDA solid medium.

2,2′-methylenebis[6-(1,1-dimethylethyl)-4-methyl-phenol] (MP) with a purity of 99% and lup-20(29)-en-3-one (lupenone, LP) with a purity of 98.0% were purchased from Shanghai Macklin Biochemical Technology Co., Ltd. (Shanghai, China). Hexane and acetone with chromatographic purity were purchased from Guangzhou Chemical Reagent Factory (Guangzhou, China).

### 2.2. The Extract Chemicals Analysis of C. rogenhoferi Nest Materials and Workers

The naturally shed part and the empty part left by the *C. rogenhoferi* nest were collected (about 2 g) and placed in a conical flask with 20 mL hexane and extracted for 6 h. After extraction, 1 mL of the extract was drawn with a disposable syringe, passed through a 0.22 μm organic filter membrane, and loaded into a 1.5 mL brown sample vial

To determine whether *C. rogenhoferi* incorporated their secretions into the nest materials, head, thorax, and abdomen extracts of *C. rogenhoferi* workers were tested. Twenty workers were divided into three parts, head, thorax, and abdomen, and each body part was pooled in 2 mL hexane for 2 h. Hexane was used as a blank sample. All samples were stored at 4 °C until analysis.

### 2.3. Gas Chromatography-Mass Spectrometry (GC-MS)

The GC-MS system consisted of an Agilent 7890B gas chromatograph equipped with an HP-5MS elastic quartz capillary column (30 m × 0.25 mm × 0.25 µm) and an Agilent 5977A mass selective detector. The chromatographic temperature program was as follows: an initial temperature of 150 °C was maintained for 1 min, and then the temperature was increased to 200 °C at a rate of 5 °C/min and held for 2 min, followed by an increase to 300 °C at a rate of 7 °C/min and held for 5 min. The injection port temperature was set at 150 °C, with an injection volume of 0.5 µL per run. The carrier gas was helium, with a flow rate of 1 mL/min, and the splitless mode was used. The mass spectrometry conditions were as follows: an EI detector with an orifice temperature of 250 °C, an ionization energy of 70 eV, and a mass range of 30–500 amu.

Compounds were identified by comparison with the reference mass spectral library NIST23. The peak area ratio was used to calculate the relative percentage of each compound based on the peak area relative to the total sample area after removing impurities detected in the blanks. Using MP standards at concentrations of 100 μg/mL, 50 μg/mL, 10 μg/mL, and 5 μg/mL, the standard curve of peak area versus concentration was derived as: y = 4779833.92 x − 6074710.74 (r² = 1.00). The amount of the target compound was determined through calculation and used as a basis for subsequent antimicrobial experiments.

### 2.4. Micro-Organisms Inhibition

The inhibition of MP and LP on *S. marcescens* and *B. bassiana* were evaluated.

The inhibition effect of MP and LP on *S. marcescens* were determined using the Bauer–Kirby agar disc diffusion assay [27]. Briefly, *S. marcescens* was uniformly applied to the LB solid medium in a petri dish (90 mm in diameter). A sterile disk sensitivity strip (6 mm in diameter) was placed in the center of the petri dish containing 10 μL of the acetone solutions of either MP or LP after 5 min air-drying. For this experiment, the test concentrations of MP were 2.53, 0.253, and 0.0253 μg/mL, and those of LP were 16.953, 1.6953, and 0.16953 μg/mL. Acetone was taken as negative control, and the blank sterile disk sensitivity strip was used as blank control. The treated petri dishes were placed into an incubator with temperature 28 °C and humidity 46%. After 24 h, the inhibition zone, which was the linear distance from the edge of the bacterial growth to the edge of the sterile disk, was measured. Two colonies were tested, and each colony was replicated three times.

The inhibition effect of MP and LP on *B. bassiana* growth were determined by the inhibition zones [28]. The method is the same as that for *S. marcescens*. The treated PDA solid medium petri dishes were placed in an incubator at a temperature of 28 °C and a humidity of 46% for 24 h before measuring the inhibition zones. For this experiment, the test concentrations of MP were 2.53, 0.253, and 0.0253 μg/mL, and those of LP were 16.953, 1.6953, and 0.16953 μg/mL. Two colonies were tested, and each colony was replicated three times.

The effects of MP and LP on the mycelium extension length of *B. bassiana* colonies were also evaluated [29,30]. PDA plates (diameter 60 mm) were streaked with *B. bassiana* under a bio-safety cabinet using an aseptic inoculation loop. A sterile disk sensitivity strip (6 mm in diameter) was placed in the center of the petri dish lid containing 10 μL of the acetone solution of either MP or LP after 5 min air-drying. The Petri dish containing the inoculated PDA plate was inverted and placed onto the lid of the Petri dish which had a treated strip. Then, the Petri dishes were sealed using parafilm “M” (Bemis^®^ Company Inc., Neenah, WI, USA) and placed in an incubator at a temperature of 28 °C and a humidity of 46%. The extension length of the mycelium of colonies were measured under microscopy after 24, 30, 36, 42, and 48 h incubation. After incubation, one 1 cm × 1 cm plug was removed from each plate randomly and observed under the EVOS FL Auto Imaging System fluorescence microscope (Carl Zeiss, Oberkochen, Germany). Average mycelium length was obtained from 20 extended mycelium taken from two randomly selected microscopic fields per replication. For the experiment, the test concentrations of MP were 2.53, 0.253, and 0.0253 μg/mL, and those of LP were 16.953, 1.6953, and 0.16953 μg/mL. Acetone was taken as negative control, and the blank sterile disk sensitivity strips were used as blank control. Each treatment was replicated three times, and the whole experiment was repeated twice.

### 2.5. Statistical Analysis

The antimicrobial activity data of tested compounds were analyzed for normal distribution using the Shapiro–Wilk test. For data that conformed to a normal distribution, a one-way ANOVA was conducted to test for homogeneity of variances. For data with homogeneous variances, a one-way ANOVA was performed, and Tukey’s test was used for multiple comparisons. For data that did not conform to a normal distribution or had unequal variances, the Kruskal–Wallis test was utilized. All data analysis procedures were executed on SPSS 26.0.

## 3. Results

### 3.1. Identification and Quantification of Chemicals Extracted from C. rogenhoferi Nests

**Figure 1:**
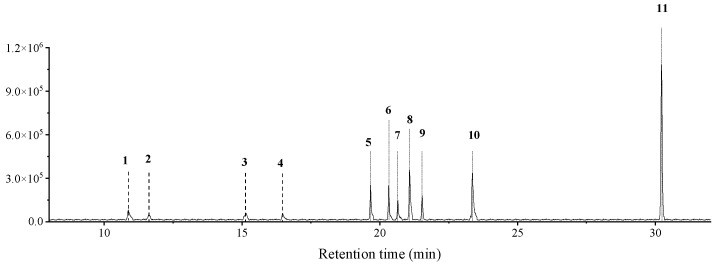
Chromatogram of chemicals extracted from nest materials of *Crematogaster rogenhoferi*. The numbers in the figure are the compound numbers in the order of peaks, corresponding to the compounds information in Table 1.

The results showed that nest materials of *C. rogenhoferi* primarily contained 11 substances: 9-octylheptane, 3-methylhexadecane, octadecane, cyclododecane, 2,2′-methylenebis[6-(1,1-dimethylethyl)-4-methyl-phenol] (MP), 1-pentene, pentane, 11-methylpentane, 2-methyltetracosane, 13-methylheptane, and lup-20(29)-en-3-one (LP) (Table 1). After peak area normalization and quantification by the external standard method, MP, which was also found in the venom and dufour’s gland of *C. rogenhoferi* (unpublished data), was present in the nest materials at a content of 5.65%, with a concentration of approximately 2.53 μg/mL. LP had the highest content at 38.01%, with a concentration of roughly 16.953 μg/mL (Figure 1).

### 3.2. Chemcial Extracts from Different Parts of Crematogaster Rogenhoferi Workers

**Figure 2:**
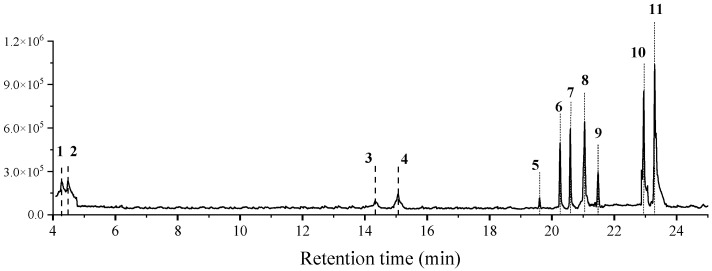
Chromatogram of extracts from the head of *Crematogaster rogenhoferi* workers. The numbers in the figure are the compound numbers in the order of peaks, corresponding to the compounds information in Table 2.

The head extract of *C. rogenhoferi* mainly contains heptadecane, 2,4-Di-tert-butylphenol, heneicosane, hocosane, MP, 1-Pentacosene, Pentacosane, 9-methyl-nonadecane, 2-Methyltetracosane, Heptacosane, 13-Methylheptacosane, and 11 other substances (Figure 2). The relative content of each substance was obtained by peak area normalization (Table 2).

**Figure 3:**
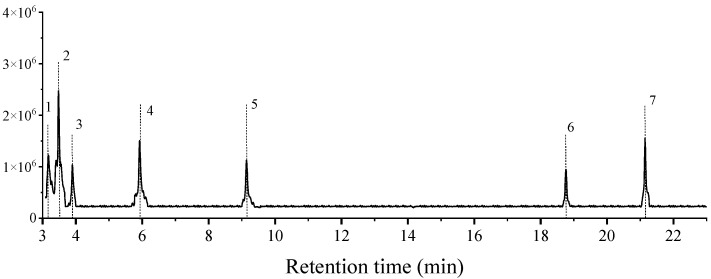
Chromatogram of extracts from the thorax of *Crematogaster rogenhoferi* workers. The numbers in the figure are the compound numbers in the order of peaks, corresponding to the compounds information in Table 3.

The extracts from the thorax of *C. rogenhoferi* workers mainly contained six substances, tetradecane, pentadecane, hexadecane, nonadecane, heptadecane, 9-octyl-, tetracosane, and hexacosane (Figure 3), and the relative contents of each substance were obtained by peak-area normalization (Table 3).

**Figure 4:**
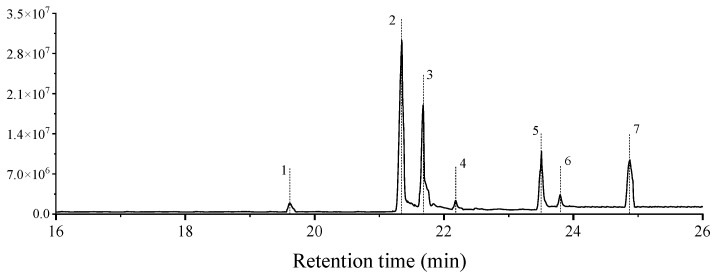
Chromatogram of extracts from the abdomen of *Crematogaster rogenhoferi* workers. The numbers in the figure are the compound numbers in the order of peaks, corresponding to the compounds information in Table 4.

The abdominal extracts of *C. rogenhoferi* workers mainly contained 2,2′-methylenebis[6-(1,1-dimethylethyl)-4-methyl-phenol], (Z)-3-(pentadec-8-en-1-yl)phenol, (Z)-3-(Heptadec-10-en-1-yl)phenol, 3-((4Z,7Z)-Heptadeca-4,7-dien-1-yl)phenol, 3-(10Z)-10-nonadecen-1-yl-phenol, 3-pentadecyl-phenol, 5-methyl-2-(4-pyridyl)- Indole, and seven other substances (Figure 4), and the relative content of each substance was obtained after peak area normalization (Table 4). Among them, 2,2′-methylenebis[6-(1,1-dimethylethyl)-4-methyl-phenol] was also detected in the nest material.

### 3.3. Inhibitory Effect of MP and LP Against Serratia marcescens

**Figure 5:**
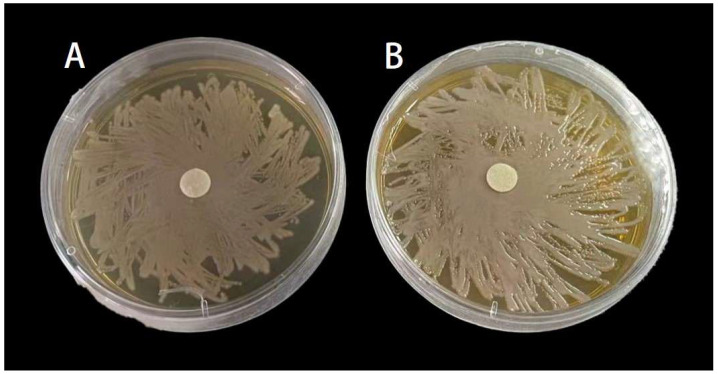
Inhibition zones of MP and LP on *Serratia marcescens*. (**A**) Treated by 2.53 μg/mL MP. (**B**) Treated by 16.953 μg/mL LP.

**Figure 6:**
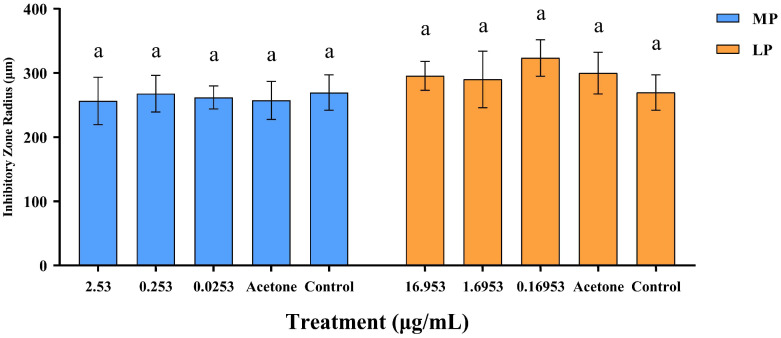
Inhibitory effect of 2,2′-methylenebis[6-(1,1-dimethylethyl)-4-methyl-phenol] (MP) and Lup-20(29)-en-3-one (LP) at different concentrations on *Serratia marcescens*. Radius of the circle of inhibition for bar data; bar values are means ± standard error of three replicates. Means with different letters are significantly different (*p* < 0.05).

MP and LP had no significant inhibitory effect on the growth of *S. marcescens* (Figure 5). The results showed that the diameter of inhibition zones in MP or LP treatment was not significantly different from that of the negative or blank control (Figure 6; For MP, *F*_4,25_ = 0.111, *p* > 0.05; For MP, *F*_4,25_ = 0.371, *p* > 0.05).

### 3.4. Effect of MP and LP Against Beauveria bassiana

**Figure 7:**
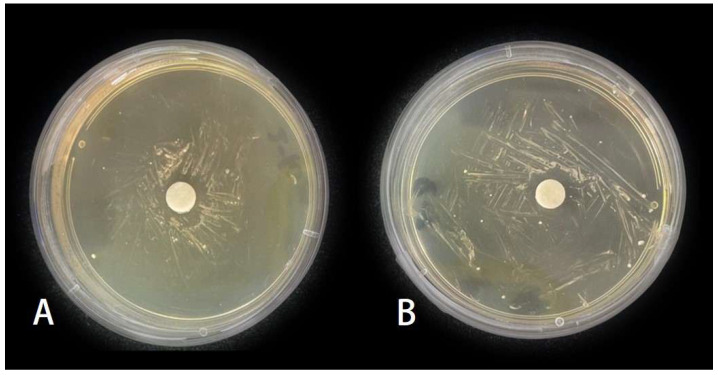
Inhibition zones of MP and LP on *Beauveria bassiana*. (**A**) Treated by 2.53 μg/mL MP. (**B**) Treated by 16.953 μg/mL LP.

**Figure 8:**
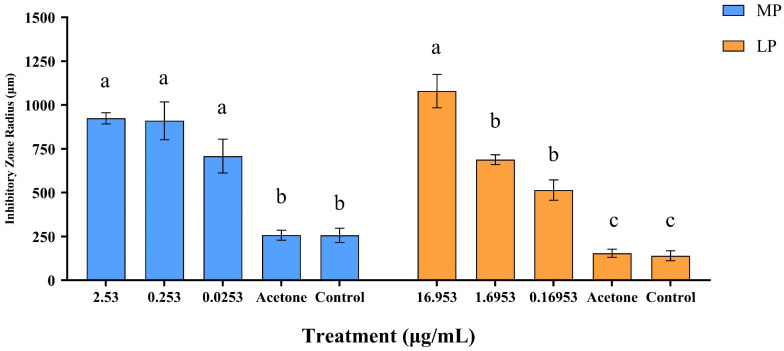
Inhibitory effect of two compounds, 2,2′-methylenebis[6-(1,1-dimethylethyl)-4-methyl-phenol] (MP) and lup-20(29)-en-3-one (LP), at different concentrations on *Beauveria bassiana* growth. Radius of the circle of inhibition for bar data; bar values are means ± standard error of three replicates. Means with different letters are significantly different (*p* < 0.05).

MP treatments can suppress the growth of *B. bassiana* (Figure 7A). The results showed that the growth of *B. bassiana* was significantly inhibited by MP (Figure 8; *F*_4,25_ = 22.978, *p* < 0.05). However, there was no significant variation in the diameter of inhibition zones among different concentrations of MP. The diameter of inhibition zones was 708.62 μm at 0.0253 μg/mL of MP treatment and 924.02 μm at 2.53 μg/mL.

LP treatments can also suppress the growth of *B. bassiana* (Figure 7B). The diameter of inhibition zones was 1079.7 μm at 16.953 μg/mL of LP treatment, which was significantly higher than those at other concentrations of LP treatments (*F*_4,25_ = 52.848, *p* < 0.05). Acetone treatment had no significant effect on the growth of *B. bassiana* (Figure 8).

### 3.5. Effects of MP and LP on the Mycelium Extension of Beauveria bassiana Through Fumigation

**Figure 9:**
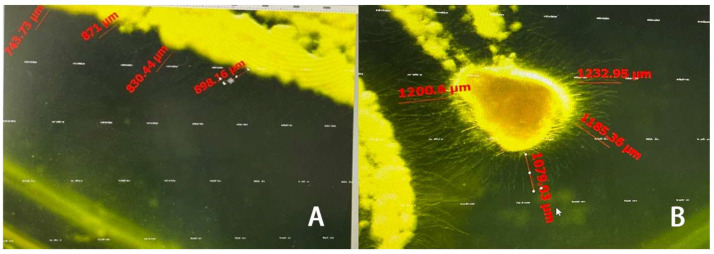
The extension length of the mycelium on *Beauveria bassiana* colonies following exposure to MP. (**A**) Treated by 2.53 μg/mL MP after 24 h. (**B**) Treated by 0 μg/mL MP after 24 h.

**Figure 10:**
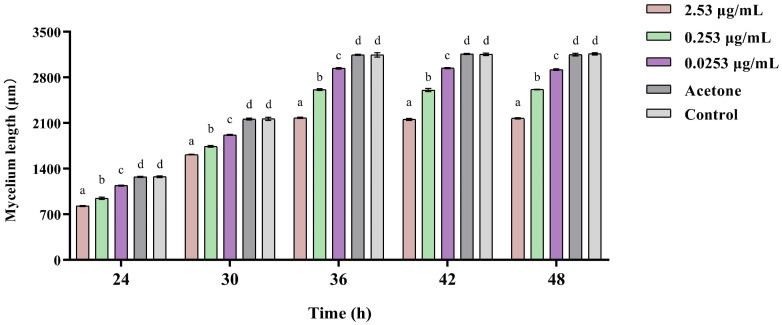
The extension length of the mycelium on *Beauveria bassiana* colonies following exposure to different concentrations of MP. Data are means ± standard error. Different letters indicate significant differences (Tukey test, *p* > 0.05).

The results showed that MP inhibited the mycelium extension of *B. bassiana* through fumigation (Figure 9) and that the higher the concentration, the more significant the inhibition effect. Within 48 h, the length of *B. bassiana* mycelium extension treated with MP was significantly lower than that of the control (Figure 10; *F*_4,25_ = 968.031, *p* < 0.05).

**Figure 11:**
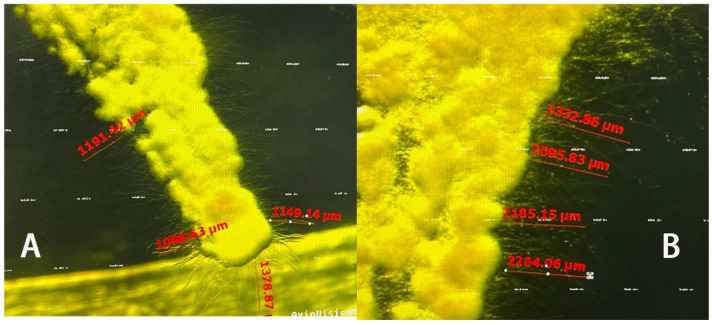
The extension length of the mycelium on *Beauveria bassiana* colonies following exposure to LP. (**A**) Treated by 16.935 μg/mL MP after 24 h. (**B**) Treated by 0 μg/mL LP after 24 h.

**Figure 12:**
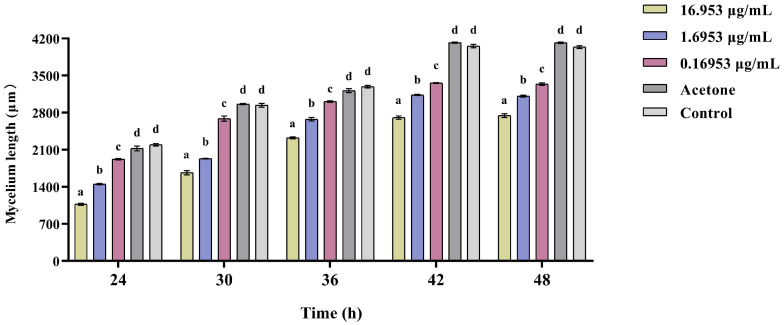
The extension length of the mycelium on *Beauveria bassiana* colonies following exposure to different concentrations of LP. Data are means ± standard error. Different letters indicate significant differences (Tukey test, *p* > 0.05).

The results showed that MP inhibited the mycelium extension of *B. bassiana* through fumigation (Figure 11), the higher the concentration, the more significant the inhibition effect. Within 48 h, the extension length of *B. bassiana* mycelium treated with MP was significantly lower than that of the control (Figure 12; *F*_4,25_ = 614.857, *p* < 0.05).

## 4. Discussion

In response to outbreaks and the spread of pathogenic microorganisms in their nests, ants have evolved appropriate collective prophylactic and defensive behaviors [31]. Reports have indicated that multiple venom alkaloids of fire ants, which have significant antimicrobial effects, have been found in soil extracts of their nests [6,32,33]. Termites secrete volatile compounds to fumigate their nests [34] and use antimicrobial feces as nesting material [35]. This study showed that LP and MP, identified from the nest materials of *C. rogenhoferi*, significantly inhibited the colony growth and mycelium extension of *B. bassiana* colonies by direct contact and fumigation. However, neither LP nor MP had any negative effect on the growth of *S. marcescens.* This suggests that *C. rogenhoferi* also applied antimicrobial chemicals to inhibit the growth of pathogens. These results showed that chemicals from nest materials may have strong inhibitory activity against fungi but weak inhibitory activity against bacteria. Research showed that ants are more sensitive to pathogenic fungi due to the greater ease of fungi growth and spread in the nests [11,31] and may have evolved a preferential defense strategy against the fungi. Another reason may be that *C. rogenhoferi* prefers to add chemicals against fungi to the nest because plant fibers of its nest are suitable for fungi growth.

Wang et al. (2015) found that fire ants *S. invicta* produce an antimicrobial property of volatiles against *B. bassiana* [12]. This study showed that MP and LP have fumigation effects on the germ tube growth of *B. bassiana*. It implied that nest fumigation may also be an important component of the social immune system in *C. rogenhoferi*. Our previous study showed that MP is an ingredient of the venom of *C. rogenhoferi*, which has repellent and contact effects on fire ants *S. invicta* (unpblished data). In this study, MP was also detected from heads of *C. rogenhoferi*. There are a variety of glands in the head of ants, the main ones reported being the prepharyngeal gland, pharyngeal gland, hypopharyngeal gland, maxillary glands etc. [36]; these glands form the salivary complex, while ant saliva components have antioxidant cleansing properties [37]. Therefore, it is hypothesized that the MP in the head of *C. rogenhoferi* may come from these glands. Chen (2007) indicated that fire ants incorporate antimicrobial agents into nesting materials to suppress the growth of pathogenic microorganisms inside the nest [38]. This phenomenon had also been reported in wasps [13,32,39]. Since MP is taken as a repellent and antimicrobial agent, we speculated that *C. rogenhoferi* may have deposited MP on nest materials protectively to protect their colony against enemies and pathogenic microorganisms.

Social animals also add fresh plant material rich in secondary volatile compounds into the nest material to render the nest anti-parasite [40]. Chapuisat et al. (2007) indicated that wood ants *F. paralugubris* mix resin from coniferous trees with their nest material to protect themselves against pathogens[18]. Ants also build their nests on plants of the *Myrmecodia* spp. group, which contain flavonoids, alkaloids, polyphenols, tannins, and saponins [41,42,43,44,45], and this type of plant has great potential as an antimicrobial drug [22]. In our study, LP was not detected from *C. rogenhoferi* workers. LP is a common secondary metabolite of plants which can be found in herbal preparations and coffee by-products [46,47], as well as in seeds, leaves, roots, and other plant tissues [48,49,50]. In the habitat of *C. rogenhoferi*, there are many kinds of plants, including *A*. *auriculiformis*, *Michelia mediocris* Dandy (Magnoliales: Magnoliaceae), *M*. *chapensis*, *Eucalyptus* spp. (Myrtales: Myrtaceae), *Cinnamomum camphora* (L.) (Laurales: Lauraceae), and other species. Since *C. rogenhoferi* build their nest relying on plants [24], the source of LP may come from the plants around *C. rogenhoferi* nests. However, despite various efforts to determine the plant information utilized for nest construction through genetic identification methods, we failed to obtain any usable DNA information from the nest material. In this study, the nests of *C. rogenhoferi* were collected from *A. auriculiformis* trees. Previous studies have shown that chemical constituents and tree extracts of *A. auriculiformis* have antifungal effects [51,52]. Although there is no report on the detection of LP in *A. auriculiformis,* derivatives and similar compounds of LP have been found in other species of the genus *Acacia* [53,54]. However, it remains to be investigated whether *C. rogenhoferi* prefer to build their nests on plants containing LP and incorporate these plant tissues into the nest material.

Excepting LP and MP, several chemicals were also found in the nest materials of *C. rogenhoferi*, including 9-octylheptane, 3-methylhexadecane, octadecane, cyclododecane, 1-pentene, pentane, 11-methylpentane, 2-methyltetracosane, 13-methylheptane, and of these, MP, 1-pentacosene, pentacosane, 2-methyltetracosane, and 13-methylheptacosane; five main chemicals were also detected from the head part of *C. rogenhoferi*. 13-methylheptacosane has previously been found in the posterior pharyngeal glands of the head of two species of harvester ants, *Pogonomyrmex salinus* Cole (Hymenoptera: Formicidae) and *Messor lobognathus* Smith (Hymenoptera: Formicidae) [55], and can also be used as a sex pheromone for the male pear woodlouse *Cacopsylla pyricola* (Forster) (Hemiptera: Psyllidae) [56]. Moreover, pentacosane and 13-methylheptacosane have both been found in the posterior pharyngeal glands of males of the solitary wasp *Philanthus Triangulum* (Fabricius) (Hymenoptera: Crabronidae) and may be stored as a marker pheromone [57]. A previous study showed that ants incorporating chemicals into the nest soil might be the basis for nest recognition [58,59,60]. It is speculated that *C. rogenhoferi* may also incorporate chemicals into its nest materials for population communication. The functions of these chemicals need to be verified in further studies.

## Figures and Tables

**Table 1:** Information on the chemicals extracted from nest materials of *Crematogaster rogenhoferi*.

Peak No.	RT (min)	Molecular Formula	Compound	Relative Content%
1	10.87	240.46	5,5-dibutylnonane	4.67
2	11.63	240.47	hexadecane, 3-methyl-	3.37
3	15.14	254.49	octadecane	3.18
4	16.47	224.43	cyclohexadecane	1.50
5	19.66	340.50	2,2′-methylenebis[6-(1,1-dimethylethyl)-4-methyl-phenol]	5.65
6	20.32	350.66	1-pentacosene	7.81
7	20.65	352.68	pentacosane	4.05
8	21.08	366.71	11-methylpentacosane	9.49
9	21.54	352.00	2-methyltetracosane	5.35
10	23.36	394.76	13-methylheptacosane	16.91
11	30.23	424.70	lup-20(29)-en-3-one	38.01

**Table 2:** Information on the substances extracted from the head of *Crematogaster rogenhoferi* workers.

Peak No.	RT (min)	Molecular Formula	Compound	Relative Content%
1	4.28	240.28	heptadecane	6.04
2	4.48	206.17	2,4-di-tert-butylphenol	8.57
3	14.33	296.34	heneicosane	3.98
4	15.07	310.36	docosane	5.17
5	19.60	340.50	2,2′-methylenebis[6-(1,1-dimethylethyl)-4-methyl-phenol]	0.56
6	20.26	350.66	1-pentacosene	8.87
7	20.59	352.68	pentacosane	9.25
8	21.04	366.71	9-methyl-nonadecane	12.80
9	21.48	352.40	2-methyltetracosane	5.18
10	22.95	380.34	heptacosane	14.48
11	23.29	394.76	13-methylheptacosane	23.80

**Table 3:** Information on the substances extracted from the thorax of *Crematogaster rogenhoferi* workers.

Peak No.	RT (min)	Molecular Formula	Compound	Relative Content%
1	3.17	198.235	tetradecane	6.88
2	3.50	212.250	pentadecane	37.71
3	3.90	226.266	hexadecane	5.67
4	5.90	268.313	nonadecane	12.69
5	9.10	352.407	9-octyl-heptadecane	12.04
6	18.76	338.391	tetracosane	6.00
7	21.14	366.423	hexacosane	8.12

**Table 4:** Information on the substances extracted from the abdomen of *Crematogaster rogenhoferi* workers.

Peak No.	RT (min)	Molecular Formula	Compound	Relative Content%
1	19.60	340.50	2,2′-methylenebis[6-(1,1-dimethylethyl)-4-methyl-phenol]	2.47
2	21.34	302.49	(Z)-3-(pentadec-8-en-1-yl)phenol	31.31
3	21.65	330.00	(Z)-3-(Heptadec-10-en-1-yl)phenol	30.40
4	22.18	328.53	3-((4Z,7Z)-Heptadeca-4,7-dien-1-yl) phenol	2.23
5	23.50	358.33	3-(10Z)-10-nonadecen-1-yl-phenol	6.07
6	23.79	304.51	3-pentadecyl-phenol	2.05
7	24.90	208.26	5-methyl-2-(4-pyridyl)-Indole	25.47

## Data Availability

The raw data supporting the conclusions of this article will be made available by the authors on request.
